# Cannabis and Suicidal Behaviour Among Adolescents: A Pilot Study from Trinidad

**DOI:** 10.1100/tsw.2005.79

**Published:** 2005-08-08

**Authors:** Hari D. Maharajh, Monique Konings

**Affiliations:** ^1^ Department of Medical Sciences, University of West Indies, MT Hope, West Indies, Jamaica; ^2^ PMS Vijverdal Psychiatric Hospital, Maastricht, the Netherlands

**Keywords:** Human development, cannabis, suicide, public health, Trinidad

## Abstract

Cannabis use and suicidal behaviour are causes of adolescent morbidity and mortality worldwide. Changing trends in these behaviours in younger age groups, higher incidence, gender differences and sociocultural variations present an enormous challenge. There is no consensus whether these complex relationships are either a direct or an indirect effect due to other mental disorders, or a social response of disclosure of drug taking habits to family members and school authorities. This paper reviews the epidemiology of suicidal behaviour and cannabis use among adolescents and looks at the relationship of these behaviours regionally and internationally. The Caribbean islands have an established use of cannabis with higher suicidal rates, which provides an ideal setting to investigate the interrelationship of these disorders. Preliminary research findings in Trinidad indicate high rates of cannabis use among school students with higher rates in vocational schools compared to grammar schools. Utilising the CAPE questionnaire, depressive and psychotic experiences were common findings in adolescent cannabis users with a significant preponderance of depressive experiences (*p*<0.01). Our findings suggest that there is a convincing relationship between suicidal behaviour and cannabis use, the latter awakening depressive experiences. Suicidal behaviour and cannabis use are major public health problems and require a multidimensional approach with culturally competent preventive interactions. School based prevention programmes are necessary at the levels of parent-teacher partnership and classroom intervention. The treatment of adolescent disorders remains a major challenge of the future. Double disorders such as cannabis use and suicidal behaviour are uncharted areas and need novel approaches.

## INTRODUCTION

Although suicidal behaviour and cannabis-related disorders are common findings in the adolescent age group, the relationship of these disorders to each other is not clear. Adolescence in itself has become a stressful transition period that requires adaptation and change in capacities and skills for maturation into adulthood. Changes in family structures, earlier sexual maturation resulting in earlier sexual drives and societal pressures on the behaviours and expectations of adolescent functioning present a formidable challenge for many young people. A vulnerable minority may complete suicide as an outcome, or attempt suicide as a coping mechanism. In addition, the use of mind-altering drugs such as cannabis may also provide respite, or solace by facilitating identification with a teenage culture, and subsequently the achievement of autonomy. While both suicidal behaviour and cannabis-related disorders can be viewed as independent coping mechanisms of maladjusted youths, it is well established that substance use increases the risk of suicidal behaviour, especially if the person is also depressed.

We wish to review the relationship between suicidal behaviour and cannabis-related disorders with emphasis on cannabis and its effect on mood disorders among adolescents. Within the Caribbean region, cultural norms of cannabis use and abuse, dosing, age of initial use, duration, tolerance and reverse tolerance and polydrug abuse may affect patterns of clinical presentations. Previously described as an innocuous drug, cannabis has a negative effect on mental health especially in young adolescents whose cerebral functioning and enduring personality have not yet fully developed.

## DEFINITIONS

Suicidal behaviour is an umbrella term, which describes suicidal ideation, or thoughts, parasuicide without intent, attempted suicide with intent and completed suicide. Parasuicide and attempted suicide are synonymous with non-deliberate self-harm and deliberate self-harm. Complete suicide, commonly referred to as suicide, is defined as a conscious act of self inflicted death. The term is ambiguous since it is based on the assumption that the person had intended to end his life when he committed the act. It is difficult to retrospectively assess the guilty mind "*mens rea*" of the victim at the time of the act. This results in inaccurate classification of an act deliberately undertaken to end ones life and accidental death. Consequently, suicide is viewed as a term that carries too much surplus meaning[1].

Cannabis related disorders incorporate cannabis use disorders of dependence and abuse and cannabis induced disorders of intoxication, delirium, anxiety and psychosis[2].The American classificatory system does not include mood disorders as an effect of cannabis use or abuse as is categorized for other psychoactive substances. The ICD-10[3] is vague in its classification. In the category of psychotic disorder (F1x.5) due to psychoactive substance abuse (F10-19), there is a clinical condition of predominantly depressive symptoms, which is applicable to all psychoactive drug use. The point is made however, that these psychotic disorders may present with varying patterns of symptoms, influenced by the type of substance involved and the personality of the user.

## EPIDEMIOLOGY

Suicidal behaviour is the third leading cause of premature death in the adolescent age group [4]. It is age dependent. The mean annual world wide rate for suicide between the ages of 5-

14 years was reported as 0.5/100.000 for females and 0.9/100,000 for males and among 15-24 years 12.0/100,000 for females and 14.2/100,000 for males[5].

Across countries, the rates of suicidal behaviour tend to vary according to ethnicity, culture, gender, state of social development, political system and religion. A comprehensive review of the US Youth Risk Behaviour Surveys, 1991-1997, reported that the number of adolescents who have contemplated suicide ranges from 29% in 1991 to 20.5% in 1997. These rates are higher than the 14.3% reported for suicidal ideation among African Americans[6]. Suicidal ideation seems to be lower in the western world. British adolescents were found to have a rate of 15%[7], while South Brazilians were reported as having a rate of 19%[8]. A higher rate of 49.6% for current suicidal ideation has been reported for adolescents from Hong Kong[9]. Significant gender differences have been reported in suicidal behaviour in Britain, France, the United States and Slovenia. In these countries, female adolescents exhibited greater levels of suicidal ideation as well as attempts[10].

Internationally, suicidal attempts range from 2.8 to 13.2%. It is lowest in Dutch adolescents with rates of 2.8% [11]. Across continents, rates of 7.3% - 7.7% were reported in American adolescents [12,13], 8.2% in Norwegian adolescents [14], 9% in South Brazilian and Chinese adolescents [6,15], 10.45 in Slovenian adolescents [11] and 13.2% in British adolescents [6].

In many Caribbean countries suicide is a major public health problem. Trinidad and Tobago has the second highest suicide rate of 11.6/100,000 (1994) only after Cuba 18.3/100,000 (1995). High rates are found in Guyana 10.5/100,000 (1994), moderate rates in St Lucia 7.5/100,000 (1988) and Barbados 6.5/100,000(1995), and low rates in the Bahamas 1.1/1000,000 (1995) and Jamaica 0.3/1000,000 (1985)[16]. A definite rate of attempted suicide is not known, but estimates range from 67-95/100.000 with attempts more common in females between the ages of 15-29[7,11,12]. A recent study of 1,845 respondents aged 14-20 in Trinidad and Tobago revealed that females had significantly higher mean Suicidal Ideation Questionnaire (SIQ) scores when compared to males (29.64 vs. 21.74) and higher rates of suicidal attempts compared to males (9.8 % vs. 4.5%[10].

## CANNABIS AND ADOLESCENTS

Cannabis use in adolescents is prevalent with its consumption increasing on a worldwide scale [17]. Two-thirds of all adolescents and young adults have had at least a single lifetime exposure to cannabis[18,19,20]. Lifetime incidence of cannabis abuse ranges from 3.5% to 8% with cannabis dependence ranging from 2.2% to 7%[20,21,22,23]. The frequency and pattern of cannabis use has increased[18] with a drop in the mean age of initial use to a mean of 12 years[22]. In Belgium, a recent study reported that the prevalence rate of weekly cannabis use increased from 1.2% in 1990 to 9.2% in 1994 and was still increasing in 1998 [21]. In two well-designed cross sectional school surveys among Norwegian students in 1992 and 2002, cannabis use increased from 5.1% to 13.5% among boys and 3.9 to 9.3% among girls. In addition, there was a significant increase in the mean score on the depression scale in both genders[17].

## CANNABIS USE IN THE CARIBBEAN

The advent of indentured workers from India to the Caribbean following the emancipation of slavery in 1838 resulted in the introduction of the plant *Cannabis indica*. Cannabis referred to as marijuana, weed, ganja, herb, hashish, bhang or pot has many ingredients, the most well known being delta-9 tetra hydrocannabinol. It is prepared from the flowers, leaves, stems and seeds of the plant, can be smoked, liquefied into teas and tonics, or grounded and baked in cookies and cakes. Its earliest use was intended for adjustment of East Indians into a new and hostile environment, separated from their loved ones thousand of miles away, oppressed by the colonial masters and socially rejected by the freed African slaves who perceived them to be strange and illiterate. It was used as a balm for solitude and quiet reflection and medicinally for stress-related disorders, asthma, pain relief, arthritis, malnutrition and other ailments.

During the post indentureship restructuring of the Indian community in 1919, cannabis was adopted as a holy weed in the religio-cultural practices of the Hindus. It found a place in Guyana and Trinidad among some seeking spiritual enlightenment and others creativity in vocal and instrumental proficiency. Later in 1930, it was incorporated into the religious practices of the newly founded Rastafarian movement in Jamaica that burrowed heavily from the habits of the indentured coolies from India.

Historically, cannabis use in the Caribbean was endemic with good social adjustment to its consumption. Until 1950, it was a legal drug that could be bought in shops and parlours. This was changed for economic and social reasons. The early pattern of collective use for recreation, enlightenment and socialization gave way to individual high quantity use for the sole purpose of ‘building a head’. This changing pattern of use also created a lucrative market for the drug trade.

Positioned between the bio-psychosocial effects of alcohol and the criminal culture of cocaine, cannabis is often viewed as a harmless drug. This perception may have emerged because of its presence for more than a century and a half with the establishment of a socially sanctioned discontinuation practice. The elders within the societies have over time determined how and by whom the drug should be used. Clear-cut prescriptions of abstinence for those with adverse effects are no longer tenable. Despite these in-built protective mechanisms, cannabis use among adolescents is on the rise with increasing adverse effects. Over the past 15 years, rates in Trinidad and Tobago have risen from 8% to 16%[23,24].

## SUICIDAL BEHAVIOUR AND CANNABIS USE

Early use of cannabis is reportedly a risk factor for depression and suicidal behaviour. In a twin study, there was a modest association between early onset cannabis use by less than 17 year old adolescents and suicidal attempts[25]. Others have reported a compelling association between heavy cannabis use and depressive disorders[26,27], with one researcher indicating that cannabis users were four times more likely to have depressive symptoms with suicidal ideation and anhedonia[28].

Not only has the age of first exposure and quantity used been implicated, but also the duration and gender of use. Brook[29] has shown that cannabis use for at least one year has resulted in social withdrawal, anxiety and depression with suicidal thoughts in adolescents, while Patton[30] has reported a five times likelihood for depression and anxiety among female adolescents and a two fold increase among male adolescents. Similar findings have been confirmed in community populations and school surveys. Teenagers who smoke cannabis on a very regular basis, at least three times per week or more, were three times more likely to have suicidal thoughts than non-users[29,31]. One US study of first graders followed into adulthood showed that early onset of cannabis use in females, but not males, signalled a modestly excessive risk of suicide attempts. Cannabis associated RR = 1.9, $p$=0.04. This group also showed an increase risk for suicidal ideation (RR = 2.9, $p$=0.006) [32]. Another finding among US high school students indicated that those who engaged in cannabis use were more likely to experience suicidal ideation and behaviour than those who abstained from cannabis[33].

The question has been asked whether cannabis abuse/dependence makes either an independent contribution to suicidal behaviour, or an indirect contribution through co-occurrence with other psychiatric and environmental factors. In a cohort of suicidal and non-suicidal patients in New Zealand 16.2% of suicidal adolescents met the DSM-111-R criteria for cannabis abuse/dependence compared to only 1.9% in the group of non-suicidal adolescents[34]. Further analysis with control of socio-demographic factors and concurrent psychiatric morbidity revealed a marginal, but significant association.

In the light of this modest strength of associations, the evidence may well reflect a link between early onset cannabis use and suicide related events[32]. The possibility remains that cannabis use may make an independent contribution to suicidal behaviour both directly and indirectly; the latter through the effects of cannabis use on the risk of other mental disorders arising from both shared genetic and ecological vulnerability.

## A PILOT STUDY

Contemporary research in Trinidad seeks to investigate patterns of clinical presentations among cannabis users in psychiatric outpatient clinics. Five clinical patterns are identified among cannabis users/abusers based on variables of dosing, age of initial use, tolerance and reverse tolerance and poly-drug abuse. These are:

- Low, controlled use with mild euphoria and heightened awareness
- Moderate use with mixed depressive symptoms and suicidal behaviour
- Heavy, short term use with manic symptoms
- Long term incremental use with psychotic symptoms (mostly negative symptoms)
- Mixed substance abuse with florid psychosis (mostly positive symptoms)

Mood disorders are not uncommon in adolescent cannabis users and may result in suicidal behaviour. Depressive symptomatology however, appears to be trumped by psychosis induced by cannabis consumption.

In an attempt to shed some light on the conundrum of depressive and psychotic symptoms induced by cannabis use, the authors are currently involved in school surveys. Preliminary findings of lifetime use and current use of cannabis in vocational schools compared to grammar schools in Trinidad revealed a greater percentile use in the former; lifetime use was 41.4% vs 19.0% and current use 23.9% vs 8.9%. (see Figure 1). There was a three-fold increase in cannabis use over a fifteen year period from 8% to 23.9 %.

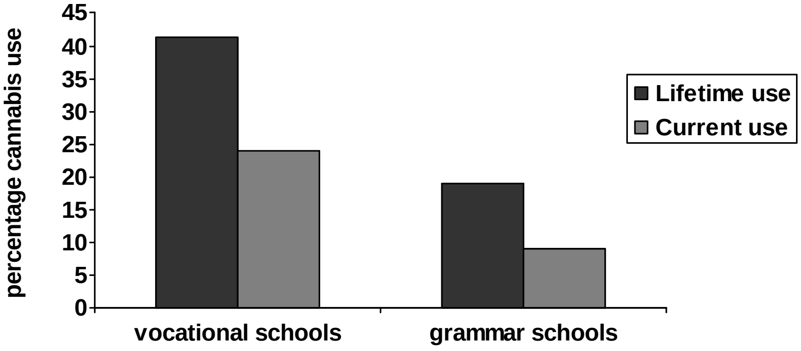

**FIGURE 1.** Rates of cannabis use in two school systems

A screening for psychiatric symptomatology was done using the Community Assessment of Psychic Experiences (CAPE) questionnaire. The CAPE questionnaire is a self-report scale that evaluates psychotic and depressive experiences and assesses the variation of positive and negative dimensions of the sub-clinical psychosis phenotype in the general population[35]. It measures the frequency of these experiences on a 4-point scale from 'never (1)', 'sometimes (2)', 'often (3)' to 'nearly always (4)'.

Our preliminary findings revealed that psychotic and depressive experiences were common findings in adolescent school children, but were increased in those using cannabis when compared to non-users. Cannabis users scored a mean of 2.039 (SD 0.922) on the depressive dimension of the CAPE compared to 1.881 (SD 0.758) for non cannabis users. This difference was statistically different ($p < 0.01$) (see Figure 2).

The mean score on the positive dimension of CAPE was 1.99 (SD 1.007) for cannabis users, and 1.81 (SD 0.817) for non users which was a statistically significant difference ($p < 0.05$). This trend was absent for the negative dimension of the CAPE analysis (see Figure 2). Variables of age of use, family history of mental illness, drug use disclosure to school authorities and family members, ethnicity, peer pressure and family structure were associated determinants of cannabis use.

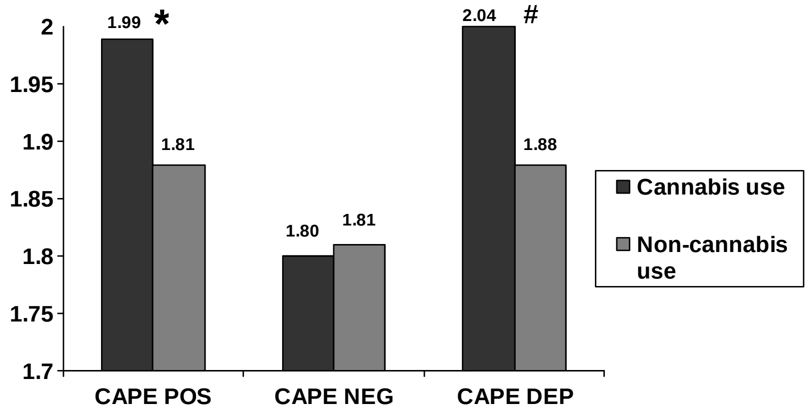

* p < 0.05 # p < 0.01

**FIGURE 2.** CAPE dimension scores in cannabis users and non-users

Age analysis of cannabis use among adolescents ages 12 to 20 (see Figure 3) shows that the peak age of current use was at age 17 with many students maturing out. This coincides with the last year of secondary schooling and graduation from vocational schools. Lifetime use increased exponentially during the adolescent period. An interesting finding was that cannabis use was higher in females at younger ages of 13 and 14.

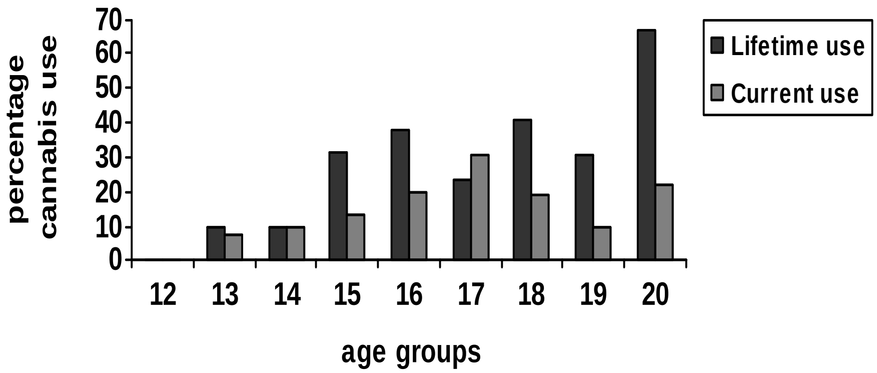

**FIGURE 3.** Current and Lifetime Cannabis use in Adolescents age 12 – 20 (n = 227).

## DISCUSSION

Suicidal behaviour with completed suicide is one of the leading causes of premature death in the adolescent age group. There is also an increase in the use of cannabis among adolescents worldwide. Both these behaviours have increased over the last decade resulting in a decline in psychosocial functioning and mental well-being. This is particularly worrisome as there is an added trend of pubertal females engaging in an increasing use of cannabis and suicidal behaviours. This effect on mental disorders in later life has been reviewed earlier.

On the background of a growing trend of the acceptance of cannabis use among Caribbean countries as an innocuous drug, a report of marijuana modified mania in Jamaica [36], a major problem in secondary schools[24], high rates of suicide in Trinidad and Guyana, and a culture invested in frolic and festivity, cannabis use has again emerged as a major public health problem. Cannabis users have enjoyed a socially protected position juxtaposed among the colourful scourges of alcoholism and cocaine use. Defences of its use as a recreational drug, medicinal herb, and holy weed albeit weak; have blinkered the negative consequences of this harmful drug. Its association with psychosis and a trigger to schizophrenia are well documented[37]. As cited in this chapter, there is compelling evidence that cannabis use is associated with depression and suicidal behaviours in the adolescent age group. The latter should be of serious concern in many Caribbean countries where rates of suicide and cannabis use are high, especially among the adolescent age group.

While the evidence for the relationship between cannabis and depression is not conclusive, it is indeed compelling in the light of its enhancement of the risk of suicidal ideation and anhedonia. It is the authors view that cannabis use trumps depression along the line of a spectrum disorder, phenomenonologically presenting with psychotic symptoms and underlying depression. This is further supported by the findings of both depressive and psychotic experiences on the CAPE questionnaire. The identification of mood disturbances in cannabis users by conventional psychometric testing is enigmatic. The CAPE questionnaire appears to be a useful tool of screening for depressive experiences that may trigger suicidal behaviour.

Our research findings in Trinidad point in the direction of cannabis use/abuse being associated with depression and suicidal behaviour. These findings are limited by the preliminary data of a small sample of 227 students. Ongoing research will provide more definitive answers. These findings support the view that there is a link between suicidal behaviour and cannabis use in adolescent. Whether there is a causal relationship or a maladaptive response to family dysfunction subsequent to disclosure of drug use has not been conclusively established. More research is needed.

Both suicidal behaviour and cannabis use are major public health problems of adolescents and are among leading causes of morbidity and mortality in developing and developed countries. School-based prevention approaches programmes aimed at health education, elucidation of risk factors, enhancing protective factors, addressing low impulse control and increasing coping mechanisms are necessary. The treatment of the double diagnosis of suicidal behaviour and cannabis use among adolescents is an uncharted area and needs a novel multidimensional approach with culturally competent preventive interactions. Interventions of motivational enhancement therapy, group cognitive behavioural therapy, family support and peer group support are needed at the levels of the parent-teacher partnership intervention and at classroom-centred intervention.

The school has a major role to play in reshaping the environment of children and teaching new skills. More training programmes are needed to increase the knowledge base of school counsellors on substance abuse, suicidal behaviours, depressive and psychotic disorders and the implementation of culturally appropriate treatment strategies. An established joint programme with the Psychiatry Unit of the University of the West Indies and Student Support Service Division of the Ministry of Education in Trinidad and Tobago aimed at training school counsellors, psychologists, supervisors and security officers in understanding, identifying, screening, treating and referring students seem to be making a difference. The provision of adequate and sustainable mental health to adolescents remains a major challenge of the future. This is the gateway towards healthy nations.

## BIOSKETCHES

**Hari D Maharajh,** BSc (Hons), MBBS (UWI), MRCPsych, Dip Clin Neuro, LLB (Hons), CMT; FRCPsych, Consultant Neuropsychiatrist, is a Senior Lecturer in the Department of Clinical Medicine at the University of the West Indies, Mount Hope, Trinidad. He has pursued an academic career with interests in medicine, law and social sciences. He studied zoology and chemistry at the University of Manitoba, medicine at the University of The Wset Indies, psychiatry at St. George's Hospital, London, and neurology at the Institute of Neurology, London. He also read law as an external student at the University of London. In 2000 he was awarded the Chaconia Gold Metal by the government of Trinidad and Tobago long and marotorious services in medicine.. He has published many scientific and popular articles and is the author of a textbook in neurology. He enjoys teaching, writing and doing research in adolescence and cultural issues. E-mail: drharim@carib-link.net.

**Monique Konings,** MD, is a postgraduate student in psychiatry. She studied medicine at the University of Maastricht, the Netherlands and did her specialization in psychiatry at PMS Vijverdal, Maastricht, the Netherlands. As partial specialization for her specialist degree, she is presently attached to the Department of Clinical Medicine at the University of the West Indies, Mount Hope, Trinidad. Her interests are research in addiction, schizophrenia and other psychosis. E-mail: koning164@zonnet.nl
